# Isoniazid (INH) mono-resistance and tuberculosis (TB) treatment success: analysis of European surveillance data, 2002 to 2014

**DOI:** 10.2807/1560-7917.ES.2019.24.12.1800392

**Published:** 2019-03-21

**Authors:** Basel Karo, Anke Kohlenberg, Vahur Hollo, Raquel Duarte, Lena Fiebig, Sarah Jackson, Cathriona Kearns, Csaba Ködmön, Maria Korzeniewska-Kosela, Dimitrios Papaventsis, Ivan Solovic, Dick van Soolingen, Marieke J. van der Werf

**Affiliations:** 1 EPIET: European Programme of Intervention Epidemiology Training, European Centre for Disease Prevention and Control, Stockholm, Sweden; 2 Field Epidemiology South East & London, National infection Service, Public Health England, London, United Kingdom; 3 Infectious Disease Department, Robert Koch Institute, Berlin, Germany; 4 These authors contributed equally to this article and share first authorship; 5 European Centre for Disease Prevention and Control, Stockholm, Sweden; 6 Directorate General of Health, Lisbon, Portugal; 7 Apopo, Sokoine University of Agriculture, Morogoro, Tanzania; 8 Health Protection Surveillance Centre, Dublin, Ireland; 9 Public Health Agency, Belfast, Northern Ireland; 10 National Tuberculosis and Lung Diseases Research Institute, Warsaw, Poland; 11 National Reference Laboratory for Mycobacteria, ‘Sotiria’ Chest Diseases Hospital, Athens, Greece; 12 National Institute for TB, Lung Diseases and Thoracic Surgery, Vysne Hagy, Catholic University Ruzomberok, Ruzomberok, Slovakia; 13 Tuberculosis Reference Laboratory, National Institute for Public Health and the Environment, Bilthoven, The Netherlands

**Keywords:** tuberculosis, TB, anti-tuberculous treatment, isoniazid mono-resistance, surveillance, epidemiology

## Abstract

Introduction: Isoniazid (INH) is an essential drug for tuberculosis (TB) treatment. Resistance to INH may increase the likelihood of negative treatment outcome.

Aim: We aimed to determine the impact of INH mono-resistance on TB treatment outcome in the European Union/European Economic Area and to identify risk factors for unsuccessful outcome in cases with INH mono-resistant TB.

Methods: In this observational study, we retrospectively analysed TB cases that were diagnosed in 2002–14 and included in the European Surveillance System (TESSy). Multilevel logistic regression models were applied to identify risk factors and correct for clustering of cases within countries.

Results: A total of 187,370 susceptible and 7,578 INH mono-resistant TB cases from 24 countries were included in the outcome analysis. Treatment was successful in 74.0% of INH mono-resistant and 77.4% of susceptible TB cases. In the final model, treatment success was lower among INH mono-resistant cases (Odds ratio (OR): 0.7; 95% confidence interval (CI): 0.6–0.9; adjusted absolute difference in treatment success: 5.3%). Among INH mono-resistant TB cases, unsuccessful treatment outcome was associated with age above median (OR: 1.3; 95% CI: 1.2–1.5), male sex (OR: 1.3; 95% CI: 1.1–1.4), positive smear microscopy (OR: 1.3; 95% CI: 1.1–1.4), positive HIV status (OR: 3.3; 95% CI: 1.6–6.5) and a prior TB history (OR: 1.8; 95% CI: 1.5–2.2).

Conclusions: This study provides evidence for an association between INH mono-resistance and a lower likelihood of TB treatment success. Increased attention should be paid to timely detection and management of INH mono-resistant TB.

## Introduction

Tuberculosis (TB) causes a large degree of suffering and an estimated 1.3 million deaths per year globally, occurring mainly in less affluent countries, but also in upper-middle and high-income countries in the European Union/European Economic Area (EU/EEA) [1,2]. In Europe, there has been a steady decline in TB notification rates of ca 5% per year. Nevertheless, TB remains a considerable problem because of multidrug-resistant (MDR) and extensively drug-resistant (XDR) TB [3].

The main public health response to the TB epidemic consists of early diagnosis, prevention of transmission and adequate treatment. In general, treatment is most successful when there is no resistance to any of the drugs designated for treatment of TB [4], and the drugs isoniazid (INH) and rifampicin (RIF) can be included in the treatment regimen. INH has long been an essential component of first-line treatment for active TB and an important drug in TB control because of its potent early bactericidal activity, low rate of adverse events and low cost [5]. Currently, there is no equivalent alternative available [6]. Resistance to INH is prevalent [4], with substantial geographic variation [7].

INH mono-resistance increases the likelihood of negative treatment outcome and progression to MDR TB [4,8,9]. Reported treatment outcome seems to differ by setting and region for cases with INH-resistant TB [10-12]. Population groups that are especially at risk of negative treatment outcome due to INH mono-resistance are children and HIV-positive patients [13-15]. Recently, two systematic reviews assessed treatment options for INH mono-resistant TB [8,16]. One review concluded that treatment with first-line drugs resulted in suboptimal outcome [8], whereas the other showed that extending the duration of RIF and increasing the number of effective drugs lowered the odds of unfavourable outcome [16]. An analysis of individual patient data conducted in the framework of a World Health Organization (WHO) guideline development process showed that the addition of a fluoroquinolone to a regimen of 6 months of daily RIF, ethambutol (EMB) and pyrazinamide (PZA) was associated with improved treatment success in INH-resistant cases [17]. After an evaluation of all available evidence, WHO has issued new guidelines on treatment for patients with INH mono-resistance [18]. Discussion is ongoing as to whether to maintain INH in the treatment regimen if a low degree of resistance is detected; however, there is limited data on the effect this strategy has on treatment outcome.

The European Surveillance System (TESSy), hosted by the European Centre for Disease Prevention and Control (ECDC), contains case-based information for more than 1.5 million TB cases reported by EU/EEA countries between 1995–2015 [3]. In contrast to the information from randomised controlled trials (RCTs) and cohort studies that were included in the systematic reviews, TESSy includes information on treatment outcome obtained in a programmatic setting [8,16]. These data are from a larger number of patients, are more recent and are more EU/EEA-focused than the data in the aforementioned reviews. We therefore set out to analyse this dataset to determine the current treatment outcome of INH mono-resistant TB in the EU/EEA and to identify risk factors for unsuccessful treatment outcome in cases with INH mono-resistance.

## Methods

### Study population and data sources

In this observational study, we retrospectively analysed TB notification data reported to the TESSy database between 2002–14. We included pulmonary and extra-pulmonary TB cases with available information on treatment outcome and drug-susceptibility testing (DST) results for at least INH, RIF, streptomycin (STR) and EMB. Information on DST for PZA was not collected in TESSy during the study period and therefore PZA was not part of our inclusion criteria.

### Operational definitions

Treatment outcome was reported 12, 24 and 36 months after the start of TB treatment. We categorised treatment outcome in accordance with the 2017 joint WHO Regional Office for Europe/ECDC surveillance and monitoring report [3]. For cases with a treatment outcome of ‘still on treatment’ at 12 or 24 months, the final treatment outcome reported at 24 or 36 months was used, respectively. Unadjusted data was stratified by age, although this stratification was not described in the protocol for this study. The operational definitions used in this study are listed in the Box.

Box **Operational definitions for analysis of European tuberculosis surveillance data, 2002–2014**

The following definitions were used:

• INH mono-resistant TB cases: cases with resistance to INH and documented susceptibility to RIF, STR and EMB;

• Cases with fully drug-susceptible TB: cases with documented susceptibility to INH, RIF, STR and EMB;

• New TB cases: cases who were never previously treated for TB or who received drug treatment for less than 1 month;

• Cases with a history of TB: cases who were previously treated for TB for 1 month or more (for countries who did not report information about previous treatment, the variable previous diagnosis was used as a proxy);

• Geographical origin of cases, i.e. native vs foreign: based on the country of birth or, if this information was unavailable, on the citizenship of the patient;

• Low TB-incidence country: country with a TB incidence rate < 10 cases per 100,000 population [31];

• High TB-incidence country: country with a TB incidence rate ≥ 10 cases per 100,000 population [31].

EMB: ethambutol; INH: isoniazid; RIF: rifampicin; STR: streptomycin; TB: tuberculosis.

### Statistical analysis and modelling approach

To investigate the impact of INH mono-resistant TB compared with fully drug-susceptible TB on treatment success, we applied a multilevel logistic regression model. To adjust for the heterogeneity between countries, the model was corrected with a random intercept at the country level for the differences in the average treatment success rate between countries and with a random slope for the differences in the INH mono-resistance effect on the treatment success rate at the country level. The necessity of adding a random intercept and a random slope in the model was determined using the likelihood-ratio test. The main outcome was dichotomised as unsuccessful treatment (failed, died, lost to follow-up and not evaluated) vs treatment success (cured or completed), using the final reported treatment outcome. Independent variables available in the TESSy data (age, sex, geographical origin, type of TB, microscopic confirmation, history of TB, HIV status and reporting year) were also assessed as possible confounders in the relationship between INH mono-resistant TB and treatment success. Independent variables that caused a change in the regression coefficient between INH mono-resistant TB and treatment success of > 10% were considered potential confounders and were retained in the final multilevel multivariable model. In addition, we evaluated the interaction of INH mono-resistant TB with age and history of TB on treatment success at a p value of 0.1. Furthermore, a sensitivity analysis was conducted to assess the impact of INH mono-resistance after excluding cases with the outcome ‘not evaluated’.

To identify risk factors for an unsuccessful treatment outcome in INH mono-resistant cases, the multilevel univariate and multivariable logistic regression models were used to examine the association between predicting variables (age, sex, geographical origin, microscopy confirmation, history of TB, HIV status, type of TB and reporting year) and unsuccessful treatment outcome. Age was dichotomised into < and ≥ the median age of the included study population. In a sensitivity analysis, cases with the outcome ‘not evaluated’ were excluded. As the outcomes were proportions, we used logistic regression for all models. Odds ratios (OR) with 95% confidence intervals (CI) were calculated to assess the strength of the association. All analyses were performed using the STATA (Stata/SE 14.1, StataCorp LP, Texas, United States (US)) software.

### Ethical statement

This study is based on data collected on the basis of statutory notification in each EU/EEA country and reported anonymously to ECDC on the basis of decision No 2119/98/EC of the European Parliament and of the Council [19]. Therefore, informed consent from patients is not required.

## Results

### Characteristics of the study population

**Figure 1:**
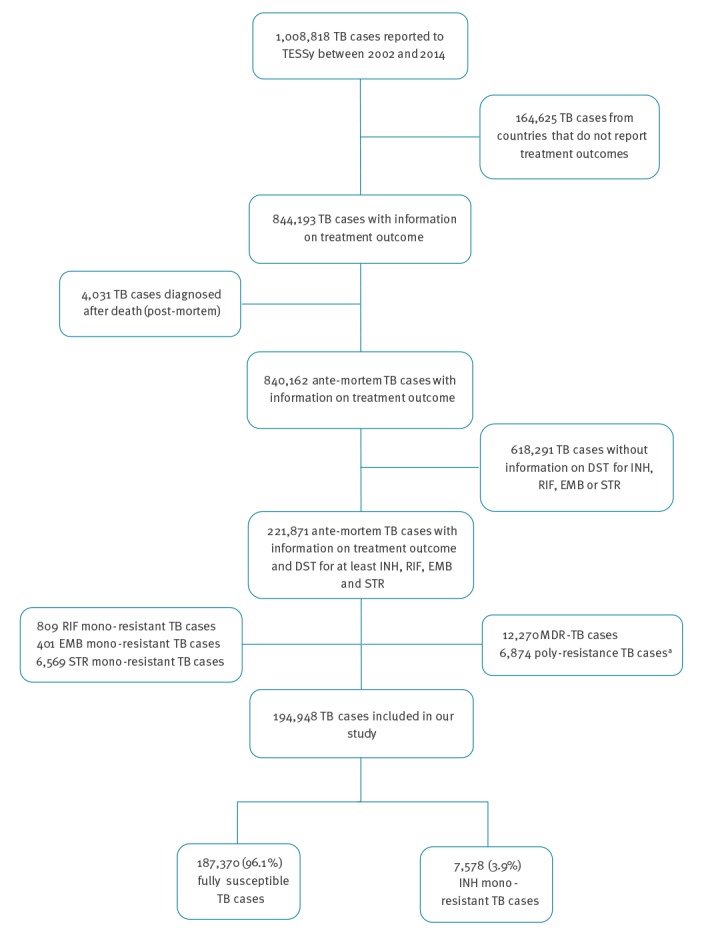
Flow chart of cases included in analysis of treatment outcome of isoniazid mono-resistant tuberculosis, 31 EU/EEA countries, 2002–2014

DST: drug-susceptibility testing; EMB: ethambutol; EU/EEA: European Union/European Economic Area; INH: isoniazid; MDR-TB: multidrug-resistant TB; RIF: rifampicin; STR: streptomycin; TB: tuberculosis; TESSy: the European Surveillance System; WHO: World Health Organization.

^a^ According to WHO, poly-resistance in TB cases refers to resistance to two or more first-line drugs, but not to both isoniazid and rifampicin, i.e. not MDR TB.

From 2002–14, a total of 1,008,818 TB cases were notified in 31 EU/EEA countries and reported to TESSy. TB cases without reported treatment outcome for the years 2002–14 (n = 164,625) or who were diagnosed post-mortem (n = 4,031) were excluded from our analysis. We also excluded 618,291 TB cases without DST results for INH, RIF, EMB or STR. Furthermore, we excluded cases with drug resistance other than INH mono-resistance (n = 26,923). The remaining 194,948 TB cases from 24 EU/EEA countries fulfilled the inclusion criteria and were therefore eligible for our analysis. Of them, 7,578 (3.9%) were cases with INH mono-resistant TB (Figure 1). Differences in the characteristics of included and excluded cases are presented in Table 1. No change in the proportion of INH mono-resistant TB was observed during the study period (Trend analyses: p value = 0.30).

Data source: TESSy.

**Table 1:** Characteristics of tuberculosis cases, by excluded and included cases, 31 EU/EEA countries, 2002–2014 (n = 1,008,818)

Characteristics	Excluded TB cases		Included TB cases		p value^a^
	n	%	n	%	
**Total**	**813,870**	**80.7**	**194,948**	**19.3**	–
Median age in years (IQR)	43 (30–58)	NA	45 (32–60)	NA	0.05
Male sex	522,843	64.3	127,642	65.5	0.91
Cases of foreign origin	172,776	22.4	56,451	29.3	0.51
Extra-pulmonary TB	177,592	21.9	28,032	14.4	0.11
New TB cases	638,026	78.4	163,546	83.9	0.02
Microscopic confirmation	296,127	48.3	93,560	61.2	< 0.01
Positive HIV status	4,976	0.6	1,248	0.6	0.68
**Proportion of cases by reporting countries**					
Austria	4,771	44.6	5,919	55.4	p < 0.01
Belgium^b^	13,830	100	0	0	
Bulgaria	14,542	71.9	5,695	28.1	
Croatia	619	38.9	972	61.1	
Cyprus	328	57.3	244	42.7	
Czech Republic	4,927	46.4	5,704	53.7	
Denmark	4,086	82.9	843	17.1	
Estonia	2,927	50.9	2,826	49.1	
Finland	2,577	58.7	1,817	41.3	
France^c^	70,140	100	0	0	
Germany	28,524	41.6	40,015	58.4	
Greece^d^	7,911	100	0	0	
Hungary	17,720	78.4	4,879	21.6	
Iceland	137	97.8	3	2.2	
Ireland	3,660	67.0	1,803	33.0	
Italy^c^	56,146	100	0	0	
Latvia	7,533	47.8	8,209	52.2	
Liechtenstein^d^	5	100	0	0	
Lithuania	13,572	51.9	12,570	48.1	
Luxembourg^d^	443	100	0	0	
Malta	305	68.9	138	31.2	
Netherlands	8,314	59.0	5,769	41.0	
Norway	1,450	34.4	2,771	65.6	
Poland	70,917	64.3	39,430	35.7	
Portugal	24,906	60.5	16,241	39.5	
Romania	311,991	98.9	3,265	1.1	
Slovenia	432	15.4	2,372	84.6	
Slovakia	4,189	52.6	3,783	47.4	
Spain^c^	54,232	100	0	0	
Sweden	5,881	82.3	1,264	17.7	
UK	76,855	73.0	28,416	27.0	

EU/EEA: European Union/European Economic Area; HIV: human immunodeficiency virus; IQR: interquartile range; TB: tuberculosis; TESSy: The European Surveillance System; UK: United Kingdom.

^a^ Obtained by a multivariable logistic regression model corrected for clustering within countries.

^b^ Not performing drug susceptibility testing for streptomycin.

^c^ Not reporting case-based drug susceptibility data and treatment outcome data.

^d^ Not reporting treatment outcome data.

Data source: TESSy.

The majority of included cases were reported by Germany, Poland and the United Kingdom (UK) (20.5%, 20.2% and 14.6%, respectively). Cases with INH mono-resistant TB were more likely to be younger than cases with fully drug-susceptible TB (median age 41 vs 46 years), be of foreign origin (37.0% vs 28.6%) and have a prior history of TB (13.6% vs 9.4%). Cases with INH mono-resistant TB were also more frequently reported by high TB-incidence countries, compared with low TB-incidence countries (65.9% vs 60.0%) (Table 2).

**Table 2:** Characteristics of tuberculosis cases by isoniazid mono-resistance status, 24 EU/EEA countries, 2002–2014 (n = 194,948)

Characteristics	Fully susceptible TB cases^a^		INH mono-resistant TB cases	
	n	%	n	%
Total	187,370	NA	7,578	NA
**Sex**				
Female	64,640	34.5	2,566	33.8
Male	122,636	65.4	5,006	66.1
Unknown	94	0.1	6	0.1
**Age (years)**				
Median (IQR)	46 (32–60)	NA	41 (30–54)	NA
**Age group**				
< 15	3,160	1.7	174	2.3
15–44	89,845	47.9	4,346	57.3
45–64	57,172	30.5	2,202	29.0
> 64	37,127	19.8	855	11.3
Unknown	66	0.1	1	0.1
**Geographical origin**				
Native	131,344	70.1	4,639	61.2
Foreign	53,648	28.6	2,803	37.0
Unknown	2,378	1.3	136	1.8
**Type of TB**				
Pulmonary	160,231	85.5	6,341	83.7
Extra-pulmonary	26,813	14.3	1,219	16.1
Unknown	326	0.2	18	0.2
**Sputum smear microscopy**				
Negative	57,063	30.4	2,183	28.8
Positive	89,898	47.9	3,662	48.3
Unknown	40,409	21.7	1,733	22.9
**History of TB**				
New TB case	157,526	84.1	6,020	79.4
Case with history of TB	17,634	9.4	1,027	13.6
Case with unknown TB history	12,210	6.5	531	7.0
**HIV status**				
Negative	17,624	9.4	823	10.9
Positive	1,203	0.6	45	0.6
Unknown	168,543	90.0	6,710	88.5
**EU/EEA countries**				
Low TB incidence	74,742	40.0	2,582	34.1
High TB incidence^b^	112,628	60.0	4,996	65.9
**Reporting years**				
2002–2005	49,532	26.4	2,089	27.6
2006–2009	61,478	32.8	2,403	31.8
2010–2014	76,360	40.8	3,086	40.6
**Number of TB cases by reporting country^c^**				
Austria	5,716	96.6	203	3.4
Bulgaria	5,416	95.1	279	4.9
Croatia	953	98.0	19	2.0
Cyprus	224	91.8	20	8.2
Czech Republic	5,626	98.6	78	1.4
Denmark	807	95.7	36	4.3
Estonia	2,721	96.3	105	3.7
Finland	1,759	96.8	58	3.2
Germany	38,700	96.7	1,315	3.3
Hungary	4,663	95.6	216	4.4
Iceland	1	33.3	2	66.7
Ireland	1,725	95.7	78	4.3
Latvia	7,648	93.1	561	7.4
Lithuania	11,790	93.8	780	6.2
Malta	136	98.5	2	1.5
Netherlands	5,508	95.5	261	4.5
Norway	2,630	94.9	141	5.1
Poland	38,579	97.8	851	2.2
Portugal	15,764	97.1	477	2.9
Romania	3,032	92.9	233	7.1
Slovenia	2,346	98.9	26	1.1
Slovakia	3,697	97.7	86	2.3
Sweden	1,204	95.3	60	4.7
UK	26,725	94.1	1,691	5.6
**Final treatment outcome**				
Treatment success	144,961	77.4	5,611	74.0
Death	14,681	7.8	516	6.8
Failed	1,111	0.6	102	1.4
Lost to follow-up	10,259	5.5	567	7.5
Not evaluated	16,358	8.7	782	10.3

EU/EEA: European Union/European Economic Area; INH: isoniazid; IQR: interquartile range; NA: not applicable; TB: tuberculosis; TESSy: The European Surveillance System; UK: United Kingdom.

^a^ TB cases susceptible to at least isoniazid, rifampicin, ethambutol and streptomycin.

^b^ High-incidence countries were defined as those with 10 or more TB cases per 100,000 population in 2015 (Bulgaria, Croatia, Estonia, Latvia, Lithuania, Poland, Portugal, Romania and the UK).

^c^ Seven countries (Belgium, France, Greece, Italy, Liechtenstein, Luxembourg and Spain) were excluded from this study, as they did not report treatment outcome and/or the required susceptibility data.

Data source: TESSy.

### Tuberculosis treatment outcome

**Figure 2:**
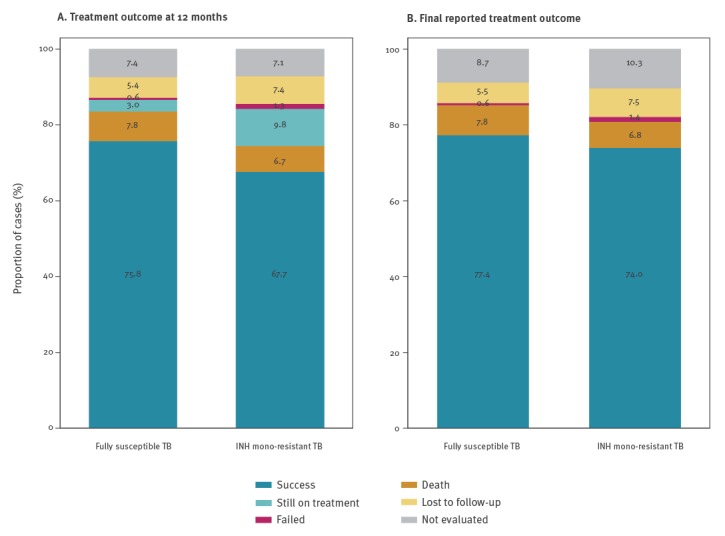
Treatment outcome of tuberculosis, by isoniazid mono-resistance status, 24 EU/EEA countries, 2002–2014 (n = 194,948)

At 12 months after the start of treatment, INH mono-resistant TB cases had a lower TB treatment success rate than cases with fully susceptible TB (67.7% (5,131/7,578) vs 75.8% (142,051/187,370)). Noticeably, a higher proportion of cases ‘still on treatment’ was observed among INH mono-resistant TB cases in comparison to fully susceptible TB cases (9.8% (745/7,578) vs 3.0% (5,651/187,370), respectively). The treatment success rate was still lower in INH mono-resistant TB cases compared with fully susceptible TB cases (74.0% vs 77.4%), when assessing the final reported treatment outcome (Table 2). Fewer INH mono-resistant cases died while being treated for TB, compared with fully susceptible TB cases (6.8% vs 7.8%) (Table 2). However, a higher proportion of cases with treatment failure was observed among INH mono-resistant TB cases in comparison to fully susceptible TB cases (1.4% vs 0.6%, respectively) (Table 2). Unknown treatment outcome (i.e. lost to follow-up and not evaluated) was documented for 17.8% of INH mono-resistant TB cases vs 14.2% of fully susceptible TB cases (Table 2, Figure 2).

EU/EEA: European Union/European Economic Area; INH: isoniazid; TESSy: The European Surveillance System.

^a^ Treatment outcome at 12 months.

^b^ Final reported treatment outcome.

Data source: TESSy.

**Figure 3:**
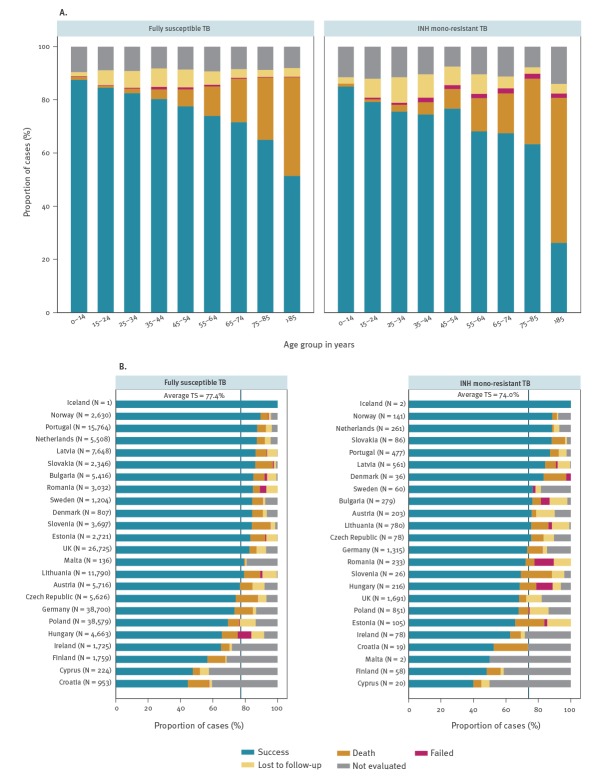
Treatment outcome of tuberculosis by isoniazid mono-resistance status and (A) age group^a^ and (B) reporting country, 24 EU/EEA countries, 2002–2014 (n = 194,948)

For cases younger than the median age of 45 years, the final treatment success rates were 76.6% (3,341/4,362) for INH mono-resistant TB and 82.6% (73,960/89,536) for susceptible TB. For cases aged ≥ 45 years, the treatment success rate decreased for both case groups; however, the difference was smaller (70.6% (2,270/3,216) for INH mono-resistant TB vs 72.6% (71,001/97,834) for fully susceptible TB). Treatment outcome by age group and INH mono-resistance status are presented in Figure 3a. The treatment success rate varied between countries for both fully susceptible and INH mono-resistant TB cases. Markedly, a relatively high proportion of deaths during TB treatment was reported among INH mono-resistant TB cases in Croatia (21.1%) and Slovenia (19.2%). In Romania and Hungary, a high proportion of cases with treatment failure was observed (12.0% and 10.2%, respectively) (Figure 3b).

EU/EEA: European Union/European Economic Area; INH: isoniazid; TB: tuberculosis; TESSy: the European Surveillance System; TS: treatment success; UK: United Kingdom.

^a^ Information missing for 67 cases.

Data source: TESSy.

### Impact of INH mono-resistance on tuberculosis treatment success

In the univariate model corrected for clustering within countries, INH mono-resistance was associated with a lower TB treatment success compared with cases with fully susceptible TB (OR: 0.8; 95% CI: 0.7–0.9). Out of all statistically evaluated covariates, adding age, microscopic confirmation or history of TB to the crude model led to the predefined change (> 10%) in the regression coefficient for INH mono-resistance and, therefore, these covariates were retained in the multivariable model as potential confounders. No interactions of INH mono-resistant TB with age (p value = 0.9) or history of TB treatment (p value = 0.2) were observed; therefore, these variables were not included in the final model.

In the final multivariable model, treatment success among INH mono-resistant TB was lower compared with fully drug-susceptible TB (adjusted OR: 0.7; 95% CI: 0.6–0.9). This corresponds to an adjusted treatment success of 74.0% for INH mono-resistant TB and 79.3% for fully susceptible TB, adjusted absolute difference of 5.3% (Supplementary Table S1). The treatment success remained lower among INH mono-resistant TB compared with fully susceptible TB in the multivariable model after excluding cases with ‘not evaluated’ as treatment outcome (OR: 0.7; 95% CI: 0.6–0.8) (data not shown).

### Factors associated with unsuccessful final treatment outcome of INH mono-resistant tuberculosis cases

**Figure 4:**
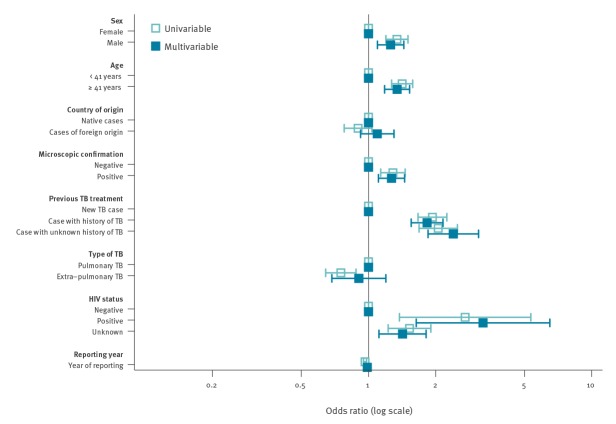
Factors associated with unsuccessful treatment among cases with isoniazid mono-resistant tuberculosis, EU/EEA, 2002–2014 (n = 7,578)

CI: confidence interval; EU/EEA: European Union/European Economic Area; INH: Isoniazid; TB: tuberculosis; TESSy: the European Surveillance System.

In the multivariable model adjusted for heterogeneity between countries, unsuccessful treatment among INH mono-resistant TB cases was associated with age ≥ median age (41 years) (OR: 1.3; 95% CI: 1.2–1.5), male sex (OR: 1.3 95%; CI: 1.1–1.4), positive microscopy (OR: 1.3; 95% CI: 1.1–1.4), history of TB (OR: 1.8; 95% CI: 1.5–2.2) and positive HIV status (OR: 3.3; 95% CI: 1.6–6.5) (Figure 4) (Supplementary Table S2). In the sensitivity analysis, excluding cases with the treatment outcome ‘not evaluated’, no change in the associated risk factors was observed (data not shown).

Univariable and multivariable analyses were based on multilevel logistic regression models corrected for clustering within countries using an unstructured covariance matrix. TB cases with available information for all predicting factors were included in the multivariable analysis (n = 5,759/7,578). Excluded cases have a slightly higher treatment success (74.7% vs 73.8%; p value = 0.46).

Data source: TESSy.

INH mono-resistant and fully susceptible TB shared the same risk factors for unsuccessful treatment, except for being of foreign origin, which was associated with a higher risk for unsuccessful treatment in fully susceptible but not INH mono-resistant TB cases in the multivariable model (data not shown).

## Discussion

Our retrospective study of European surveillance data, including 7,578 cases of INH mono-resistant TB and 187,370 cases of fully susceptible TB, shows that INH mono-resistance is associated with lower TB treatment success in the final TB treatment outcome. This association between INH mono-resistance and lower treatment success is in line with a previous systematic review [20]. Although this review was published in 2009, it includes many studies that were published before the year 2000 and were mainly conducted in countries in Asia and Africa. The same applies to the reviews evaluating treatment regimens for INH mono-resistant TB that were published in 2016 [16] and 2017 [8], respectively. Therefore, our analysis of more recent 2002–14 EU/EEA surveillance data represents an addition to the currently available evidence.

Studies of TB treatment outcome under routine programmatic conditions have shown inconsistent results regarding treatment outcome of INH mono-resistant TB. While studies from the US [10], Denmark [12] and Israel [21] reported treatment outcome for INH mono-resistant TB as excellent, highly successful or similar to drug-susceptible TB, studies from Peru [22], Mexico [23], Georgia [24] and South Africa [11] showed poorer treatment outcome compared with fully susceptible TB. Possible explanations might be differences in the included patient populations; for example, regarding HIV prevalence [11,12], the availability of resources for patients, the accessibility of healthcare systems or the use of different treatment regimens. Our study, which pools data from 24 low HIV-prevalence and predominantly high-income European countries, shows that INH mono-resistance negatively affects treatment outcome. The study findings also highlight the need for timely identification of patients with INH mono-resistant TB, especially as rapid testing in recent years has focused more on the detection of RIF resistance as a proxy for MDR TB [6].

Comparison of studies has been hampered by different definitions used for INH mono-resistance. While many studies retained all INH resistance profiles, provided that RIF resistance and therefore MDR TB was excluded [8,16], other studies required additional documented susceptibility for STR and EMB [23] or analysed INH mono-resistant and INH poly-resistant cases with additional resistance to STR or EMB separately [12]. To avoid misclassification, we have used the stricter definition requiring documented DST results for INH, RIF, STR and EMB for all cases. However, while gaining specificity in the definition of INH mono-resistance, this approach has resulted in the loss of a large number of cases for which these susceptibility testing results were not available, with considerable differences in the percentages of cases that could be included by country.

The finding that EU/EEA countries have different proportions of patients with INH mono-resistant TB still under treatment at 12 months might be a surveillance artefact related to variation in reporting procedures or the result of the use of different treatment regimens with different durations, reflecting the current lack of an agreed standard regimen for the treatment of INH mono-resistant TB cases [18,25,26]. As the 12-month outcome therefore did not seem to be the most adequate endpoint for analysis of the treatment outcome of INH mono-resistant TB, we chose a composite outcome for this study using the final documented outcome irrespective of the time to reporting (12 months, 24 months or 36 months after the start of treatment).

The factors found to be associated with a higher risk of unsuccessful treatment outcome in INH mono-resistant TB in this study–higher age, male sex, positive microscopy, positive HIV status–have been described before as associated with unsuccessful TB treatment outcome independent of drug resistance status [27,28]. Prior TB treatment has also been reported as a risk factor for unsuccessful outcome in patients with INH mono-resistant TB [29]. In our study, these factors influenced TB treatment outcome regardless of the presence or absence of INH mono-resistance, indicating that they are not specific to INH mono-resistant TB, but rather are associated with lower TB treatment success in general.

The strengths of our study are the inclusion of a large number of cases and the application of a multilevel model to correct for TB clustering. This allowed to control for possible selection bias related to reporting countries and for unobserved heterogeneity between countries, thereby enhancing the generalisability of our findings. However, there are also several limitations to our study, mainly related to the use of surveillance data collected with the aim to inform TB programme management and not to evaluate clinical outcomes. As a result, data on the severity of TB disease, underlying diseases and treatment regimens were not available.

In addition, information on DST for PZA is not available in the TESSy data. In a sensitivity analysis using German notification data (40,063 TB cases, of which 1,310 were INH mono-resistant) that include information on DST for PZA, cases resistant to PZA were more frequent among INH mono-resistant TB compared with otherwise fully susceptible TB cases (5.4% vs 1.8%; p < 0.01). However, no impact of the PZA resistance status on the relationship between INH mono-resistant TB and treatment success was observed (adjusted OR: 0.8; 95% CI: 0.6–1.4; p = 0.77). Another limitation related to DST is that no information is available on the level of INH-resistance and the type of INH resistance mutations involved, which have been shown to influence treatment outcome [30].

Lastly, our study included data from only 24 of 31 EU/EEA countries; cases from seven countries were excluded due to lack of reporting treatment outcome or case-based susceptibility data (Table 1). Therefore, our data pertain only to these 24 EU/EEA countries and cannot be generalised to the whole EU/EEA without caution. Of note, 80.7% of reported cases had to be excluded due to missing information. As systematic reasons for lack of reporting within the 24 countries with included cases are not known, it is not possible for us to hypothesise how this might have affected our findings.

In conclusion, this study shows that treatment of patients with INH mono-resistant TB under routine programme conditions leads to lower treatment success compared with fully susceptible TB. The association of INH mono-resistance with negative treatment outcome highlights the need to pay increased attention to the timely identification and management of these cases to ensure treatment success for individual patients, as well as to reduce the risk for further resistance development on a population level.

## Supplementary Data

496000 Supplementary Material S1
