# Supplementary material for: Isoniazid (INH) mono-resistance and tuberculosis (TB) treatment success: analysis of European surveillance data, 2002 to 2014
Source: Euro Surveill. 2019 Mar 21;24(12):1800392. doi: 10.2807/1560-7917.ES.2019.24.12.1800392 (PMC6440580; doi:10.2807/1560-7917.ES.2019.24.12.1800392)
Supplement: Supplementary Material S1 [file 18-00392_KOHLENBERG_Supplementary_material.pdf]

SUPPLEMENTARY MATERIAL

This supplementary material is hosted by *Eurosurveillance* as supporting information alongside the article "Isoniazid (INH) mono-resistance and tuberculosis (TB) treatment success: analysis of European surveillance data, 2002 to 2014" on behalf of the authors who remain responsible for the accuracy and appropriateness of the content. The same standards for ethics, copyright, attributions and permissions as for the article apply. *Eurosurveillance* is not responsible for the maintenance of any links or email addresses provided therein."

Supplementary Table 1. Multilevel logistic regression model of the impact of isoniazid mono-resistance on the treatment success of tuberculosis in 24 European Union and European Economic Area (EU/EEA) countries, The European Surveillance System (TESSy) 2002–2014.

Independent variables	Univariate analysis		Multivariable analysis	
	**OR**	**95% CI**	**OR**	**95% CI**
Resistance status (exposure of interest)				
Fully susceptible TB	1		1	
INH mono-resistant TB	0.8	0.7-0.9	0.7	0.6- 0.9
Sex				
Female	1			
Male	0.7	0.7-0.8		
**Age**				
**Increase in age per year (continuous variable)**	**0.9**	**0.9-0.9**	**0.9**	**0.9-0.9**
Country of origin				
Native cases	1			
Cases of foreign origin	1.2	1.1-1.2		
**Microscopy confirmation**				
**Negative**	**1**		**1**	
**Positive**	**0.9**	**0.8-0.9**	**0.9**	**0.8-0.9**
**History of TB**				
**New TB case**	**1**		**1**	
**Case with history of TB**	**0.6**	**0.5-0.6**	**0.6**	**0.5-0.6**
**Case with unknown history of TB**	**0.5**	**0.4-0.5**	**0.5**	**0.5-0.6**
Type of TB				
Pulmonary TB	1			
Extra-pulmonary TB	1.1	1.1-1.1		
HIV status				
Negative	1			
Positive	0.4	0.3-0.4		
Unknown	0.7	0.7-0.8		
Year of reporting				
Years from 2002 to 2014 (continuous variable)	0.9	0.9-0.9		

Outcome coding: unsuccessful treatment=0; treatment success=1.
The model is corrected with both a random intercept and a random slope for INH mono-resistant TB at the country level using an unstructured covariance matrix.
Independent variables that caused a change in the regression coefficient of INH mono-resistant TB (exposure of interest) of >10% were considered as potential confounders (**in bold**) and included in the final multilevel multivariable model.
Interactions of INH mono-resistant TB with age and history of TB treatment on treatment success were not significant at a p-value of 0.1 (0.9 and 0.2, respectively), therefore the interactions terms were not included in the multivariable model.
OR: odds ratio; CI: confidence interval; TB: tuberculosis; INH: isoniazid.

Supplementary Table 2. Multilevel logistic regression model of factors associated with unsuccessful treatment among isoniazid mono-resistant tuberculosis cases in 24 European Union and European Economic Area (EU/EEA) countries, The European Surveillance System (TESSy) 2002–2014.

**Independent variable**	**Univariate analysis**		**Multivariable analysis***	
	**OR**	**95% CI**	**OR**	**95% CI**
Sex				
Female	1		**1**	
Male	1.3	1.2 - 1.5	**1.3**	**1.1-1.4**
Median age				
<41 years	1		**1**	
≥41 years	1.4	1.3 - 1.6	**1.3**	**1.2-1.5**
Country of origin				
Native cases	1		1	
Cases of foreign origin	0.9	0.8 -1.0	1.1	0.9-1.3
Microscopy confirmation				
Negative	1		**1**	
Positive	1.3	1.1 - 1.4	**1.3**	**1.1-1.4**
History of TB				
New TB case	1		**1**	
Case with history of TB	1.9	1.7 - 2.2	**1.8**	**1.5-2.2**
Case with unknown history of TB	2.0	1.7 - 2.5	**2.3**	**1.8-3.1**
Type of TB				
Pulmonary TB	1		1	
Extra-pulmonary TB	0.7	0.6 - 0.9	0.9	0.7-1.2
HIV status				
Negative	1		**1**	
Positive	2.7	1.4 - 5.3	**3.3**	**1.6-6.5**
Unknown	1.5	1.2 - 1.9	**1.4**	**1.1-1.8**
Year of reporting				
Years from 2002 to 2014 (continuous variable)	0.9	0.9-0.9	0.9	0.9-1.0

Outcome coding: unsuccessful treatment=0; treatment success=1.
The model is corrected with both a random intercept and a random slope for INH mono-resistant TB at the country level using an unstructured covariance matrix.
*Cases with available information for all predicting factors were included in the multivariable analysis (N= 5,756/7,578). Excluded cases have slightly higher treatment success (74.7 vs. 73.8; *P*-value 0.46).

OR: odds ratio; CI: confidence interval; TB: tuberculosis; INH: isoniazid.
